# Detailed Kinetics of the Direct Allo-Response in Human Liver Transplant Recipients: New Insights from an Optimized Assay

**DOI:** 10.1371/journal.pone.0014452

**Published:** 2010-12-29

**Authors:** Özlem Tapirdamaz, Shanta Mancham, Luc J. W. van der Laan, Geert Kazemier, Kris Thielemans, Herold J. Metselaar, Jaap Kwekkeboom

**Affiliations:** 1 Department of Gastroenterology and Hepatology, Erasmus MC University Medical Center, Rotterdam, The Netherlands; 2 Department of Surgery, Erasmus MC University Medical Center, Rotterdam, The Netherlands; 3 Laboratory of Molecular and Cellular Therapy, Vrije Universiteit Brussel, Brussels, Belgium; New York University, United States of America

## Abstract

Conventional assays for quantification of allo-reactive T-cell precursor frequencies (PF) are relatively insensitive. We present a robust assay for quantification of PF of T-cells with direct donor-specificity, and establish the kinetics of circulating donor-specific T cells after liver transplantation (LTx). B cells from donor splenocytes were differentiated into professional antigen-presenting cells by CD40-engagement (CD40-B cells). CFSE-labelled PBMC from LTx-recipients obtained before and at several time points after LTx, were stimulated with donor-derived or 3^rd^ party CD40-B cells. PF of donor-specific T cells were calculated from CFSE-dilution patterns, and intracellular IFN-γ was determined after re-stimulation with CD40-B cells. Compared to splenocytes, stimulations with CD40-B cells resulted in 3 to 5-fold higher responding T-cell PF. Memory and naïve T-cell subsets responded equally to allogeneic CD40-B cell stimulation. Donor-specific CD4^+^ and CD8^+^ T-cell PF ranged from 0.5 to 19% (median: 5.2%). One week after LTx, PF of circulating donor-specific CD4^+^ and CD8^+^ T cells increased significantly, while only a minor increase in numbers of T cells reacting to 3^rd^ party allo-antigens was observed. One year after LTx numbers of CD4^+^ and CD8^+^ T cells reacting to donor antigens, as well as those reacting to 3^rd^ party allo-antigens, were slightly lower compared to pre-transplant values. Moreover, CD4^+^ and CD8^+^ T cells responding to donor-derived, as well as those reacting to 3^rd^ party CD40-B cells, produced less IFN-γ. In conclusion, our alternative approach enables detection of allo-reactive human T cells at high frequencies, and after application we conclude that donor-specific T-cell PF increase immediately after LTx. However, no evidence for a specific loss of circulating T-cells recognizing donor allo-antigens via the direct pathway up to 1 year after LTx was obtained, underscoring the relative insensitiveness of previous assays.

## Introduction

After allogeneic transplantation, recognition of major allo-antigens by recipient T-cells occurs via two different pathways: 1) the direct pathway whereby intact donor MHC is presented by donor-derived Antigen-Presenting Cells (APC) to recipient T cells; 2) The indirect pathway, whereby processed donor MHC is presented to recipient T cells as peptides on self-MHC molecules expressed on self-APC [1], [2].

Direct pathway T cells are activated by donor-derived APC that migrate from the graft into recipient secondary lymphoid tissues [3], [4], [5], [6]. Animal studies have shown that donor APC migration is a transient phenomenon after transplantation [3], [5]. Recently, we observed that a similar transient migration of donor-derived APC into recipients occurs after liver transplantation (LTx) in humans [7]. Therefore, the general presumption is that direct pathway responses dominate during the early post-transplant period, but subside thereafter [2]. However, due to their cross-sectional approach [8], [9], [10], [11], [12], [13] not any study has described the kinetics of recipient T-cell alloreactivity after organ transplantation in humans in detail.

A common assumption is that frequencies of allogeneic T cells stimulated by the direct pathway are 100- to 1000-fold higher than responses to pathogens [2], [14]. Animal studies showed that frequencies of T cells with direct allo-specificity range between 0.01 and 21% [15], [16], [17], [18], [19], [20], [21]. However, the reported alloreactive T cell frequencies in humans are generally lower, ranging from 0.001 to 0.1% [8], [12], [13], [22], [23]. This raises the question if this difference in reported frequencies is due to an underestimation of alloreactive T cells in humans caused by suboptimal sensitivity of conventional assays.

Traditionally, allo-reactive T cells have been quantified by limiting dilution analysis (LDA). It is now known that LDA detect frequencies of pathogen-specific T cells that are one to two logs lower than those detected by MHC tetramer staining [24], [25]. Hence, a more robust assay for quantification of alloreactive human T cells is required. Because the restricted availability of MHC-tetramers (especially of MHC class II tetramers) is insufficient to cover the enormous heterogeneity of HLA, and the knowledge of peptides involved in direct pathway allo-recognition is limited [26], we chose an alternative approach.

The first aim of the present study is to set up a sensitive assay for quantification of frequencies of direct pathway alloreactive T cells. For this purpose, we used CD40-activated donor-derived B cells instead of donor leukocytes from spleen or blood, as stimulator cells, and calculated frequencies of responding recipient T-cells from division patterns measured by flow cytometric analysis of carboxyfluorescein succinimidyl ester (CFSE) fluorescent dye dilution. CFSE-dilution has a sensitivity similar to MHC-tetramer staining [27] [28], [29], [30], and CD40-B cells are a uniform source of professional APC [31], [32], [33], [34]. The second aim was to determine the kinetics of the direct pathway allo-response after liver transplantation by applying this assay.

## Materials and Methods

### Ethics Statement

The Ethics committee of the Erasmus Medical Center has approved the collection of blood in patients after liver transplantation for immunologic monitoring. A written consent from patients was obtained prior to the study.

### Patients

The study of the longitudinal kinetics of the direct allo-response included 13 patients who underwent an orthotopic liver transplantation between 1997 and 2000 at Erasmus University Medical Centre, and had stable graft function up to 1 year after LTx. All patients received a graft from a deceased donor. Indication for LTx was: post-alcoholic liver cirrhosis (n = 4), primary sclerosing cholangitis (n = 4), chronic hepatitis C (n = 1), chronic hepatitis B (n = 1), auto-immune hepatitis (n = 1), Morbus Wilson (n = 1), and heamangio-endothelioma (n = 1). In addition, we measured the allo-response in 5 additional patients before and at 1 year after LTx. Indications for LTx in these patients were: primary sclerosing cholangitis (n = 1), chronic hepatitis B (n = 2) and polycystic liver disease (n = 2). Initial immunosuppressive therapy consisted of cyclosporine A (CsA) or tacrolimus together with prednisone, and with or without azathioprine. Patient follow up was at least one year.

### Patient and donor cells

Splenic tissue from donors was obtained during multi-organ donation procedure, and single cell suspensions were made. Venous blood from patients was collected before transplantation and at several time points after transplantation. For optimization of the assay conditions, blood from healthy individuals was obtained from the blood bank. Peripheral Blood Mononuclear Cells (PBMC) and splenocytes were isolated by Ficoll-Hypaque density gradient centrifugation and stored frozen in 10% DMSO solution.

### CD40-B cell generation

B cells were expanded from donor splenocytes or recipient PBMC using a mouse fibroblast cell line stably transfected with human CD40L (L-CD40L) that was kindly provided by prof. Cees van Kooten (LUMC, Leiden, The Netherlands) [35]. L-CD40L cells were irradiated (52 Gy) and plated on 6-well plates (Costar, Cambridge, USA) at a concentration of 0.2×10^6^ cells per well in medium containing RPMI 1640 (Lonza, Basel, Switzerland), 10% heat inactivated FCS (Sigma-Aldrich, St Louis, US) and 1% penicillin/streptomycin (Gibco, California, USA). After overnight culture, L-CD40L cells were rinsed with RPMI. PBMC were seeded at 1×10^6^ cells/ml IMDM (Lonza) with 10% heat inactivated human male AB serum (Lonza), 1% penicillin/streptomycin and 1% insulin-transferrin-selenium solution (Gibco) (B-cell medium) on L-CD40L cells. Recombinant human interleukin-4 (rhIL-4) (40 IU/ml) (Strathmann Bioscience, Germany) and CsA (1 µg/ml) (Novartis, Basel, Switzerland) to prevent T-cell expansion were added. Every 3–4 days the cultured cells were transferred to freshly irradiated L-CD40L cells in a ratio of 1 CD40L cell: 4 CD19^+^ cells. Proportions of B- and T cells were checked at regular intervals. When the percentage CD19^+^ cells was >85%, CsA was discontinued for at least 3 days before their use in T-cell stimulators. When the purity of CD19^+^ cells was >95%, CD40-B cells were used in assays.

### CFSE-MLR

Before labeling with CFSE (Invitrogen, Paisley, UK), PBMC were thawed and recovered during overnight culture at 37°C with 5% CO_2_. Labeling was performed with 0.5 µM CFSE. CFSE-labeled PBMC (1×10^5^) were stimulated with 2×10^5^ irradiated (30 Gy) donor CD40-B cells, third party CD40-B cells, or autologous PBMC-derived CD40-B cells in 96-wells U-bottom plates in a final volume of 200 µl B-cell medium. Each assay was performed in triplicate. PHA-stimulated recipient PBMC were included as positive controls to test viability of thawed PBMC. Results were only included if PBMC showed responsiveness to PHA. Unless otherwise stated in the results section, flow cytometric analysis was performed after 6 days of culture at 37°C and 5% CO_2_. In quadruple experiments of the additional 5 patients, T cells were restimulated at day 5 of culture with donor-derived or 3^rd^ party-derived CD40-B cells for 24 hours, and during the last 15 hours Brefeldin A (Sigma) was added to enable measurement of IFN-γ production.

### Data Analysis with ModFit® Software

**Figure 1:**
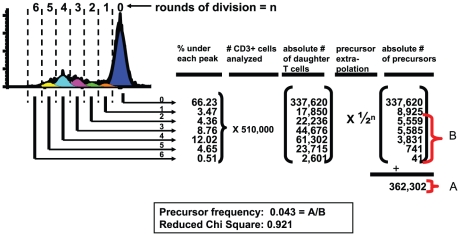
Analysis of CFSE-dilution patterns by ModFit® software. ModFit-derived CFSE-pattern of CD3^+^ T cells after 6 days of stimulation with allogeneic CD40-B cells. The software draws peaks within the CFSE-histogram centered on halving intensity values from the parental peak CFSE-intensity. Non-divided T cells are CFSE^high^ and are depicted blue, while the peaks left from it correspond to divided cells. Based on the enumerated proportions of T cells detected in each generation ( = % under each peak) and the total number of CD3^+^ T cells analyzed by the flowcytometer, the program calculates the absolute numbers of daughter T cells in each generation. The numbers of precursors which gave rise to the daughter cells are extrapolated by dividing the absolute numbers of T cells in each generation by 2^n^, in which n stands for the division cycle ( = absolute # of precursors). The PF is calculated by dividing the numbers of precursors from generation 2 onwards ( = B) by the total number of precursors in all generations including the parent peak and generation 1 ( = A). In this example the calculated PF =  (362,302 – (337,620 + 8925)/362,302) ×100 = 4.3%. The fit of the derived Gaussian peaks in the CFSE-dilution pattern is indicated by the reduced chi-square value. Values below 5 were considered as a good fit according to the manufacturer's instructions, and were included in the analysis.

Precursor frequencies were calculated using ModFit LT® software (Verity Software House, USA). The basic principles of the calculation model of the program are that the cell number duplicates at each division and that CFSE is equally distributed over the daughter cells, resulting in a two-fold reduction of the CFSE intensity after each division cycle [36] as explained in Figure 1. The software calculates backwards the *precursor frequency (PF)*, which is the proportion of the total cells calculated to have been present at the start of the experiment that responded to the allo-stimulus by dividing. Because the width of the intensity histogram of T cells in the parent generation spreads slowly during culture, it is difficult to differentiate cells that have divided once from non-divided cells. Supported by other studies [28], [37], we considered cells that had undergone at least two divisions as responders in our data analysis. Only ModFit® plots with a reduced chi-square (which is a measurement for the association of the calculated model and the real CFSE-dilution histogram: or in other words, a measurement for the fit of the model into the dilution histogram) smaller than 5 were included in the analysis. Mean PF were calculated from triplicate cultures. To assess the accuracy of the technique, we calculated the coefficient of variation of triplicates in 6 patients using the following formula: SD_triplicate_/mean _triplicate_.

### FACS data acquisition

Cultured cells were stained for 15 minutes at room temperature in 96-wells U-bottom plates with appropriate concentrations of CD3-PE (Biolegend, San Diego, USA), CD4-APC (eBioscience, San Diego, US) and CD8-Pacific Blue (BD Biosciences, San Jose, CA, USA) antibodies in a final volume of 50 µl PBS. In individual experiments indicated in the Results section, cultured cells were stained with CD8-Pacific Blue mAb, after which cells were fixed and permeabilized with Fix & Perm® reagent (ADG Bio Research GmbH, Vienna, Austria) and incubated with PE-labeled anti-granzyme B (Sanquin, Amsterdam, The Netherlands) and anti-perforin (BD Pharmingen) mAb to analyze intracellular expression of cytotoxic molecules. 7AAD (BD Biosciences, San Jose, CA, USA) was added just before measurement started to exclude dead cells. In experiments in which T cells were re-stimulated with donor-derived or 3^rd^ party-derived CD40-B cells during the last 24 hours of culture, intracellular IFN-γ was measured upon permeabilization with Fix & Perm® (An der Grub, Vienna, Austria) reagent and labeling with PeCy7-conjugated anti-IFN-γ (eBisocience, San Diego, US). Data acquisition was performed with a Canto II Flowcytometer from BD Biosciences (San Jose, CA, US).

### FACS sorting

PBMC from healthy subjects (200×10^6^) were labeled in PBS with sterile CD14-PE (BD Biosciences, San Jose, US), anti-BDCA1-PE (Miltenyi, Bergisch Gladbach, Germany), CD19-PE, CD123-PE, CD56-PE, CD45RA-FITC and CD45RO-APC (all from Beckman Coulter, Miami, US) in a final volume of 500 µl for 30 minutes at 4°C, followed by a double wash step with 3 ml PBS (Lonza). The cell pellet was resuspended to a concentration of 50×10^6^ PBMC/ml PBS. Naïve and memory T-cell populations were sorted with a FACS Aria (BD Biosciences, San Jose, CA, USA) by exclusion of PE-positive cells and gating on the CD45-RA^high^ and CD45-RO^high^ cells. CD3^+^ purity was checked after sorting with CD3-PerCP-Cy5 (Biolegend, San Diego, US).

### IFN-γ ELISPOT

LTx-recipient PBMC (2×10^5^) were pre-incubated in a 12-wells culture plate at 37°C and 5% CO_2_ with 4×10^5^ splenocyte-derived CD40-Bcells in a final volume of 600 µl B-cell medium. After 48 hours cells were harvested, washed twice with PBS and re-suspended in 200 µl of B-cell medium. Cells were transferred to two Elispot wells coated with anti-IFN-γ antibody, and incubated in a final volume of 100 µl per well for 5 hours at 37°C. Subsequently, the Elispot assay (U-Cytech Biosciences, Utrecht, The Netherlands) was executed according the manufacturers protocol. Spot analysis was performed with a Bioreader 3000 Elispot reader (Biosys, GmbH, Karben, Germany). Because of preparation-induced cell death, we based our frequency calculations on the hypothesis that eventually 5×10^4^ recipient PBMC were transferred to each of the two coated Elispot wells.

### Statistics

Differences between groups were analyzed using the Wilcoxon test and were considered statistically significant if the p-value was ≤0.05.

## Results

### CD40-B cells expand and differentiate from splenocytes and PBMC into professional antigen presenting cells

**Figure 2:**
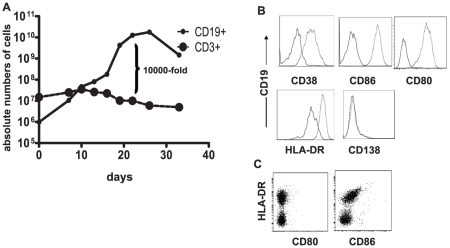
Expansion and differentiation of B cells using L-CD40L cells. A. Human splenocytes or PBMC were co-cultured with L-CD40L cells, IL-4 and CsA. Within 3 weeks, a 10^4^ fold increase of the numbers of B cells was obtained with a purity of >95%. T cells did not expand due to the presence of CsA. The current graph represents expansion from PBMC, splenocytes gave similar results. B. Cell surface marker expression on CD40-B cells harvested at day 12 of culture. CD40L-expanded B cells were activated (CD38^+^) and expressed the co-stimulatory molecules CD80 and CD86, and MHC class-II, indicating that they became professional APC. However, they did not become plasma cells, since they lacked CD138. Solid lines represent the expression of surface markers at day 0; dotted lines represent the expression at day 12 after culture. C. Donor splenocytes contained about 50% of HLA-DR^+^ APC, however these lacked CD80 and showed low expression of CD86.

Within 21 days, co-culture of human splenocytes or PBMC with L-CD40L cells in the presence of IL-4 and CsA resulted in a 10^4^-fold increase of total B-cell numbers, followed by a decrease after 27 days (Figure 2A). The decrease was due to loss of CD40 expression on CD40-B cells (data not shown). B-cell purity >95% was reached between day 9 and 12. CD40-B cells did not only expand, but also differentiated to professional APC, expressing CD38 (activation marker), HLA-DR and co-stimulatory molecules (Figure 2B). Contrary, human splenocytes contained 47±5% HLA-DR+ APC, of which only a small fraction expressed co-stimulatory molecules (CD80+: 0.9±0.9%, CD86+: 7.1±5.2%; n = 6) (Figure 2C), indicating that CD40-B cells are a more uniform preparation of APC. CD40-B cells did not differentiate to plasma cells, as all cells lacked CD138.

### Optimization and validation of CFSE-MLR

**Figure 3:**
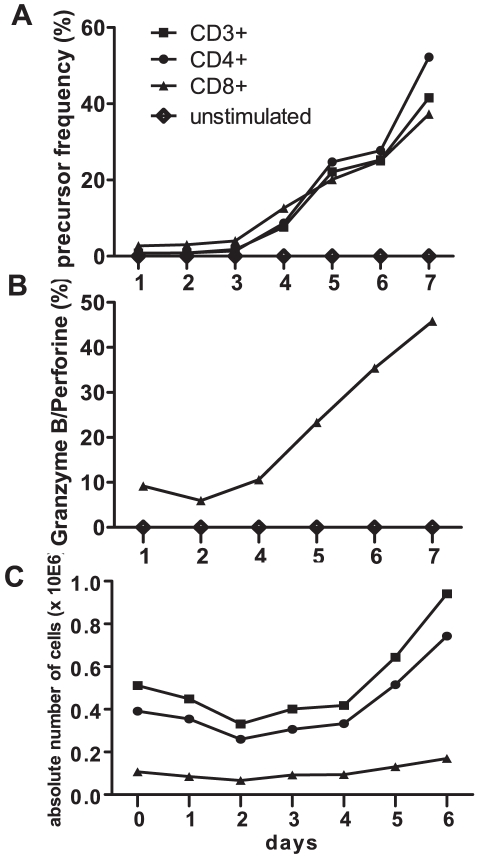
Kinetics of allogeneic T-cell responses to CD40-B cell stimulation. CFSE-labeled PBMC from a healthy individual were cultured for 7 days with allogeneic splenocyte-derived CD40-B cells. A. CFSE-dilution patterns of CD3^+^, CD4^+^, CD8^+^ T cells were analyzed daily, and PF were calculated with ModFit® software. B. Intracellular granzyme B and perforin expression were analyzed daily in CD8^+^ T cells. Depicted are the percentages of CD8^+^CFSE^low^ T cells that expressed granzyme B and/or perforin. C. Absolute numbers CD3^+^, CD4^+^ and CD8^+^ T cells during co-culture of CFSE-labelled PBMC with allogeneic CD40-B cells. Vital PBMC were counted daily using trypan blue, and proportions of CD3^+^, CD4^+^ and CD8^+^ T cells were analyzed by flowcytometry. Absolute numbers of T-cell subsets were calculated by multiplying the number of vital PBMC with the proportions of T cells obtained from flowcytometric analysis.

To determine the optimal culture period for quantification of T cells reacting to allogeneic stimulation with CD40-B cells, CFSE-dilution patterns of CD3^+^, CD4^+^ and CD8^+^ cells were analyzed daily till day 7 of culture using ModFit® software. Proliferation of T cells started at day 4, and new T cells were recruited to proliferate until day 5, thereafter PF reached a plateau value (Figure 3A). After day 6 PF increased again, which is probably due to non-specific bystander activation [27]. Therefore, we decided to determine PF at day 6 of culture. CD8^+^ T cells acquired cytolytic molecules from day 4 onward, and at day 7 about 50% of CD8^+^ T-cells that proliferated in response to allogeneic CD40-B cells ( = CFSE^low^ CD8^+^ T cells) expressed granzyme B and/or perforin, indicating that they had acquired cytolytic effector function (Figure 3B).

In calculating PF, ModFit® assesses the total number of T cells at the start of the allogeneic stimulation using backward calculation from the cells analyzed at the end of the culture. In contrast, LDA and ELISPOT calculate PF based on the numbers of T cells that are put into culture. To assess whether this difference would influence the outcomes of the different approaches of calculating PF, we counted daily the numbers of vital PBMC and calculated the proportions of CD3^+^, CD4^+^ and CD8^+^ T cells in CD40-B cell-stimulated CFSE-MLR. Figure 3C shows that numbers of all T-cell subsets decreased during the first 2 days of cultures by 36% for the CD3^+^, 34% for the CD4^+^ and 38% for the CD8^+^ T cells, a phenomenon called preparation-induced cell death [38]. Thereafter, numbers of T cells increased. Most likely, T cells alive at day 2 are the real starting population from which allo-reactive T cells are recruited to proliferate, suggesting that LDA and ELISPOT underestimate PF, while CFSE-based assays may yield more accurate PF.

### Comparison of CD40-B cells with conventional splenocytes as stimulators in CFSE-MLR and comparison of CFSE-MLR with IFN-γ ELISPOT

**Figure 4:**
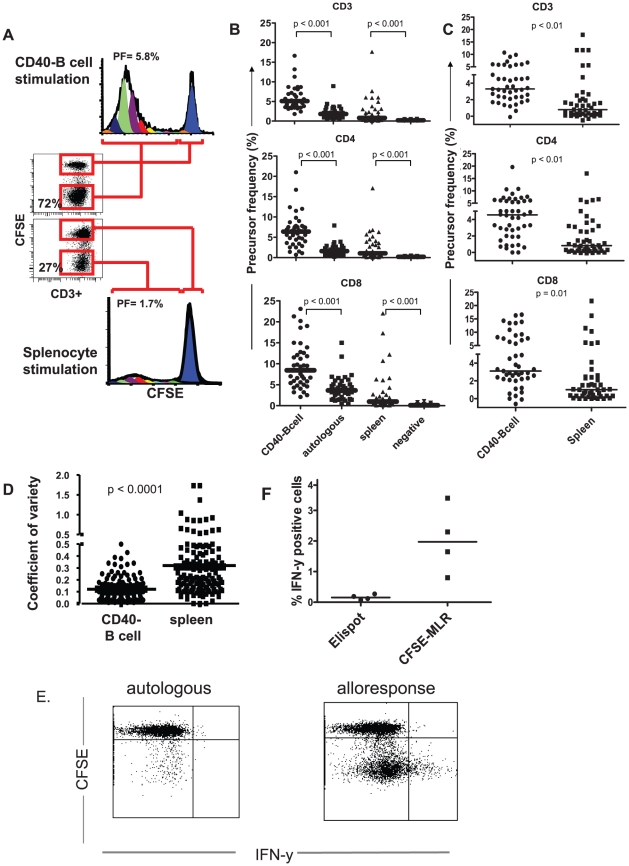
Comparison of CD40-B cell and splenocyte stimulation and comparison of CFSE-MLR with IFN-γ ELISPOT. A. CFSE-labeled PBMC from an LTx-recipient were stimulated for 6 days with allogeneic splenocyte-derived CD40-B cells or with splenocytes from the same donor. Dot-plots and CFSE-dilution graphs of CD3^+^ T cells show increased T-cell proliferation upon stimulation with CD40-B cells compared to splenocytes. Percentages in dot plots depict % dividing (CFSE^low^) T cells. Percentages in histograms are calculated PF. B: Paired comparisons of PF of LTx-recipient CD3^+^, CD4^+^ and CD8^+^ T cells upon stimulation with donor-derived CD40-B cells, autologous patient CD40-B cells, splenocytes or without stimulus. Sixty CFSE-labelled PBMC-samples of 6 LTx-recipients, obtained either before, or at different time points after transplantation, were stimulated with donor-derived or autologous CD40-B cells or with splenocytes from the same donor for 6 days. Bars represent median values. C: Donor-specific T-cell responses obtained by subtraction of PF reacting to autologous CD40-B cells from PF responding to donor-derived CD40-B cells, or subtraction of PF in the absence of allo-antigen (negative controls) from PF responding to donor splenocytes. Bars represent median values. D: Paired comparisons of the coefficients of variation of triplicate measurements of LTx-recipient CD3^+^ PF in 108 PBMC-samples of 6 LTx-recipients upon stimulation with donor-derived CD40-B cells or donor-derived splenocytes. Bars represent median values. E. Flowcytometry dotplot showing CFSE-dilution versus intracellular IFN-γ of CD3+ T cells stimulated with autologous or allogeneic CD40-B cells, and re-stimulated at day 5 for 24 hours with CD40-B cells of the same donor, the last 15 hours in the presence of Brefeldin A. Only T cells that divided (CFSE^low^) in response to allogeneic CD40-B cells produce IFN-γ. F. Comparison of CFSE-MLR with IFN-γ ELISPOT. Four different CFSE-labeled PBMC samples from LTx-recipients were stimulated for 5 days with allogeneic splenocyte-derived CD40-B cells, and re-stimulated for 24 hours with CD40-B cells of the same donor to detect intracellular IFN-γ expression. In parallel, samples of the same PBMC were analyzed for IFN-γ production upon stimulation with the same CD40-B cells in IFN-γ ELISPOT. IFN-γ-producing cells in ELISPOT are depicted as percentage of PBMC, while the percentages of IFN-γ producing cells in CFSE-MLR are depicted as percentages of CD3+ T cells.

To compare the efficacy of CD40-B-cells with conventional splenocyte stimulation, CFSE labeled PBMC from patients were co-cultured with donor splenocyte-derived CD40-B cells or with non-manipulated splenocytes from the same donor. Figure 4A displays that at day 6 T cells responded abundantly to stimulation with CD40-B cells, while splenocyte stimulation resulted in a much lower response. The results of paired comparisons between stimulations of LTx recipient PBMC with CD40-B cells or splenocytes are summarized in Figure 4B and C. Although stimulation with autologous CD40-B cells also resulted in T-cell proliferation, responses to allogeneic CD40-B cells were significantly higher (4B). The PF of T cells specifically responding to donor-derived CD40-B calculated in this ways were 6.8 fold, 5.9-fold and 8.6-fold higher PF for CD3+, CD4+ and CD8+ T cells, respectively, compared to specific responses to donor-derived splenocytes. Specific allogeneic responses to donor-derived CD40-B cells were calculated by subtraction of PF reacting to autologous CD40-B cells, and specific allogeneic responses to donor splenocytes by subtraction of PF of unstimulated T cells (4C). These data also reveal that comparable proportions of alloreactive precursors are detected within CD4^+^ and CD8^+^ subsets upon CD40-B cell stimulation (median PF: 6.4% ±4.0 for the CD4+ and 8.45% ±5.4 for the CD8+ subpopulation). Finally, CD40-B cell stimulation resulted in less variation between triplicate PF measurements than splenocyte stimulation; the coefficient of variation of measured triplicates was 3-fold lower (Figure 4D).

To enable comparison of CFSE-MLR with IFN-γ ELISPOT, at day 5 of culture we re-stimulated recipient PBMC for 24 hours with CD40-B cells of the same donor, added Brefeldin A during the last 15 hours, and measured intracellular IFN-γ expression at day 6. Interestingly, IFN-γ was only produced by T cells that had divided, but not all dividing cells produced IFN-γ (Figure 4E). In parallel, samples of the same PBMC were analyzed for IFN-γ production upon stimulation with the same CD40-B cells in IFN-γ ELISPOT. The percentages IFN-γ-producing CD3+ T cells detected in CFSE-MLR were considerably higher than the percentages of IFN-γ producing cells detected in ELISPOT, showing that CFSE-MLR is more sensitive to detect IFN-γ producing T cells upon allogeneic stimulation than ELISPOT (Figure 4F).

### Relative contributions of naïve and memory T cells to the alloresponse measured by CFSE-dilution

**Figure 5:**
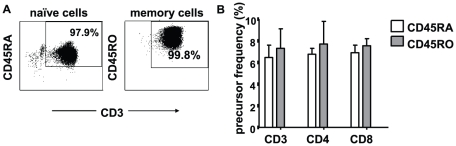
Relative contributions of naïve and memory T cells to the allo-response in CFSE-MLR upon stimulation with CD40-B cells. CFSE-labelled naïve CD3^+^CD45RA^+^and CD3^+^CD45RO^+^ memory T cells were isolated from PBMC of healthy individuals by flowcytometric sorting, and stimulated with allogeneic CD40-B cells for 6 days. A. Purity of CD3^+^CD45RO^+^ and CD3^+^CD45RA^+^ T cells after sorting. B. PF of CD3^+^CD45RO^+^ and CD3^+^CD45RA^+^ T cells proliferating upon stimulation with allogeneic CD40-B cells. Depicted are means ± SD of data from two independent experiments, each with 3 replicates.

To dissect the contributions of naïve (CD45RA^+^) or memory (CD45RO^+^) T cells to the allogeneic responses measured by CSFE-MLR upon stimulation with CD40-B cells, we sorted CD3^+^CD45RA^+^ and CD3^+^CD45RO^+^ cells (Figure 5A) and stimulated them separately with CD40-B cells. Figure 5B shows that after 6 days of culture memory and naïve subsets contributed equally to the alloresponse, with median CD3^+^ T-cell PF of 6.7% and 7.7%, respectively.

### Kinetics of the direct pathway donor-specific T-cell response after LTx

**Figure 6:**
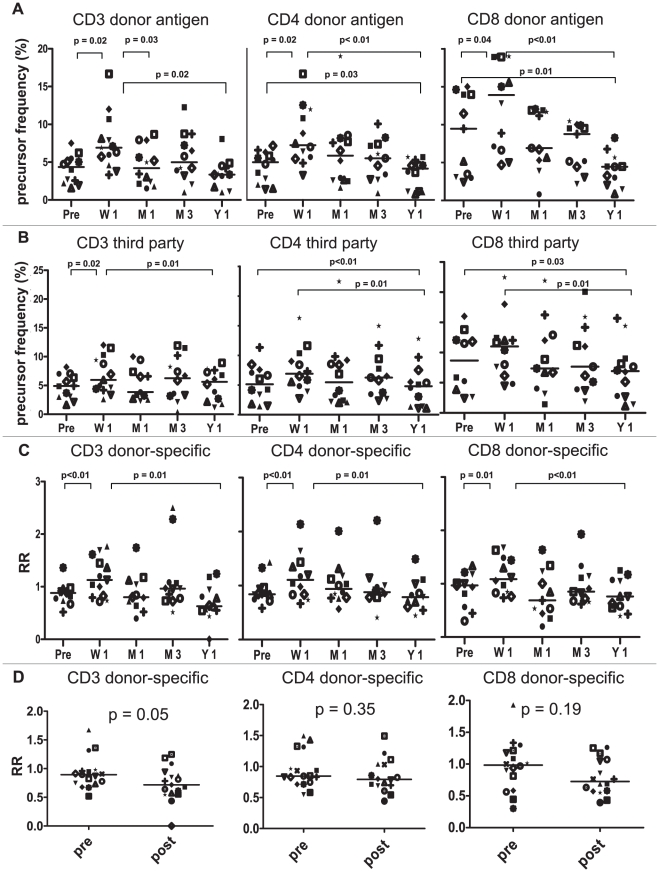
Longitudinal course of circulating donor-specific T-cell precursor frequencies in LTx-recipients. CFSE-labeled PBMC of 13 LTx-recipients obtained before transplantation (Pre), or at 1 week (W1), 1 month (M1), 3 months (M3) or 1 year (Y1) after transplantation, were co-cultured for 6 days with donor-derived splenic CD40-B cells, 3^rd^ party splenic CD40-B cells or autologous PBMC-derived CD40-B cells. PF of CD3^+^, CD4^+^ and CD8^+^ T cells responding to these stimulators were determined. Recipients and donors differed on the average in 1.5 HLA-AB alleles and in 1.7 HLA-DR alleles. Third-party stimulators were mismatched with recipients in 1.7 HLA-AB alleles and 1.8 HLA-DR alleles. Donor-derived and 3^rd^ party-derived stimulator cells differed on the average at 1.8 HLA-AB loci and 1.5 HLA-DR loci. A. Numbers of T-cell precursors responding to donor-derived CD40-B cells increased significantly 1 week after transplantation in all T-cell subsets, followed by a decrease to values below pre-transplant levels. B. Non-specific variations in allo-responses were determined by stimulating patient PBMC with third-party spleen-derived CD40-B cells. Changes in PF of CD4^+^ and CD8^+^ T cells responding to 3^rd^ party CD40-B cells between subsequent time points were generally smaller compared to those in donor-specific PF. C. Donor-specific responses were calculated by dividing PF responding to donor-alloantigen by third-party PF to obtain the relative responses (RR). RR increased significantly 1 week after transplantation in all T-cell subsets, followed by a significant decrease at 1 year after LTX. D. Comparison of relative CD3^+^, CD4^+^ and CD8^+^ T-cell responses (RR) of 18 LTx-recipients before and 1 year after LTx. Five additional patients were analyzed, and their data were added to the data of the 13 patients shown in C. Donor-derived and 3^rd^ party-derived stimulator cells used in assaying the 5 additional patients were fully MHC mismatched.

To determine the kinetics of donor-specific T cell reactivity after LTx, we quantified PF of donor-specific T cells in PBMC of 13 LTx-recipients with stable graft function at 1 year after LTx, using donor-spleen derived CD40-B cells as stimulators. PBMC were collected before transplantation, and 1 week, 1 month, 3 months and 1 year after transplantation. To evaluate the specificity of variations in responses to donor allo-antigens, we stimulated CFSE-labeled patient PBMC also with 3^rd^ party spleen-derived CD40-B cells or with CD40-B cells derived from autologous PBMC. In all samples measured, PF of T cells reacting to autologous CD40-B cells were low compared to PF of T cells reacting to donor or 3^rd^ party CD40-B cells. In addition, autologous responses did not change over time after transplantation. On the contrary, the numbers of circulating T cells reacting to donor-derived CD40-B cells increased significantly at 1 week after LTx in all T-cell subsets, followed by a decrease to values below pre-transplant levels 1 year after LTx (Fig. 6A). Variations in T-cell PF reacting to 3^rd^ party allo-antigens showed the same trend, although the differences in PF between subsequent time points were generally smaller, and the increases in the first week after LTx were not significant in the separate CD4^+^ and CD8^+^ T-cell subsets (Figure 6B). To correct variations in the response to donor allo-antigens for nonspecific changes in numbers of circulating allo-reactive T cells, we calculated the donor-specific responses (relative response; RR) by dividing PF to donor antigens by PF to third parties. Figure 6C shows that the RR significantly increased in all T-cell subsets during the first week after LTx compared to the pre-transplant levels (CD3^+^: 1.4-fold, CD4^+^: 1.3-fold and CD8^+^: 1.1-fold), followed by a gradual decrease after week 1. However, after correction for responses to 3^rd^ party allo-antigens, none of the donor-specific responses declined below pre-transplant values.

In contrast to these data, conventional assays have generally shown that stable organ transplant recipients develop donor-specific T-cell hypo-responsiveness [8], [9], [10], [11], [13], [22], [39]. To ensure that the our aberrant observation was not due to a limited patient sample size, we analyzed donor-specific and third-party T-cell responses in 5 additional LTx-recipients before LTx and 1 year after LTx. When the RR-values of all LTx-patients analyzed (n = 18) before and 1 year after LTx were compared, again no evidence for CD4^+^ and CD8^+^ donor-specific T-cell hypo-responsiveness at 1 year after LTx was obtained, although there was a tendency to slightly lower donor-specific total CD3^+^ T-cell numbers 1 year after LTx (Figure 6D).

**Figure 7:**
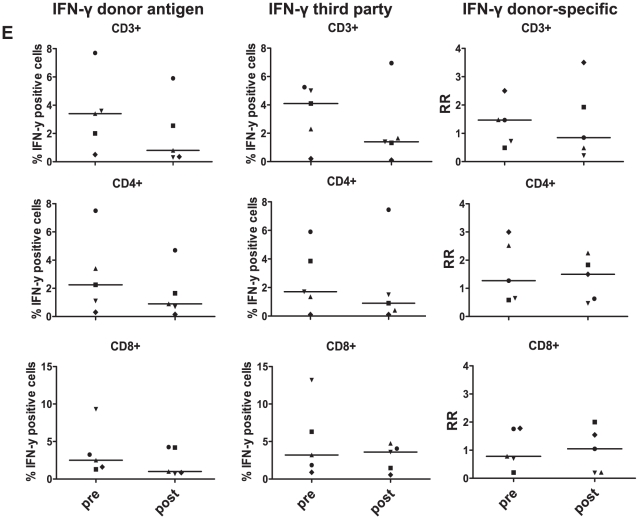
Comparison of IFN-γ production by T cells in CFSE-MLR before and 1 year after LTx. CFSE-labeled PBMC from 5 LTx-patients were stimulated with donor-derived or 3^rd^ party CD40-B cells, and re-stimulated with the same allo-antigens at day 5 of culture for 24 hours. During the last 15 hours Brefeldin A was added, and CFSE-dilution and intracellular IFN-γ was determined at day 6. Depicted are the percentages of T cells producing IFN-γ in response to donor-derived CD40-B cells and 3^rd^ party-derived CD40-B cells, and the RR of IFN-γ producing T cells. No statistically significant differences were observed in comparing RR at 1 year after LTx versus before LTx (p≥0.44).

Finally, we compared effector functions of T cells reacting to donor- and 3^rd^ party allo-antigens by measuring intracellular IFN-γ in 5 LTx-recipients before and 1 year after LTx. For this purpose we re-stimulated T cells at day 5 of culture with donor-derived or 3^rd^ party-derived CD40-B cells for 24 hours and added during the last 15 hours Brefeldin A. The proportions of T cells producing IFN-γ in response to donor allo-antigens were decreased 1 year after LTx compared to pre-LTx values. However, the same was true for IFN-γ responses to 3^rd^ party allo-antigens, resulting in similar RR of IFN-γ producing cells in all T-cell subsets before and 1 year after LTx (Figure 7). Together these data show a donor-specific increase in direct pathway T cell PF immediately after LTx, followed by a non-specific decline of allo-reactive T cells numbers to values slightly below pre-transplant levels within the first year after LTx, but no specific loss of circulating donor-specific T-cell clones.

## Discussion

Combining CFSE-dilution as a read out with CD40-B cells as stimulator cells, we developed a robust assay that detects human T cells with direct allospecificity at frequencies comparable to those predicted by animal experiments and 1- to 3-logs higher than PF detected with conventional LDA or ElLISPOT [19], [20], [21], [40], [41]. This CFSE-MLR assay is able to discriminate between CD4 and CD8 PF and detects both naïve and memory T-cell responses to allo-antigens in the same culture. By application of this assay we found that, even with immunosuppressive therapy, numbers of circulating CD4^+^ and CD8^+^ T cells with direct donor-specificity increase immediately after LTx, indicating that both subsets are primed by the graft, followed by a gradual decrease within the first year. Importantly, after correction of changes in responses to 3^rd^ party allo-antigens, no specific loss of donor-specific T cells was observed, showing that CD4^+^ and CD8^+^ T cells with direct donor-specificity persist in the circulation up to at least 1 year after LTx. However, a non-specific decrease in frequencies of circulating allo-reactive CD4+ and CD8+ T cells to values slightly below pre-transplant values was observed 1 year after LTx, a well as a non-specific decrease in their capacity to produce IFN-γ.

The first improvement contributing to the robustness of the described assay is utilizing CD40-B cells as stimulators, resulting in 3- to 5-fold higher PF compared to stimulations with splenocytes. CD40-engagement induces B-cell differentiation into professional APC with uniform expression of co-stimulatory molecules, that are able to prime both memory and naïve T-cell reponses [31], [34], [42], [43], [44] [45]. Contrary, splenocytes contain only about 50% APC, of which a minority express co-stimulatory molecules. CD40-B cells can be expanded from thawed splenocytes or PBMC, even when these have a low viability, and their expansion enables repeated measurements of donor-specific T-cell reactivity in cases of limited supply of donor cells. Importantly, the absence of T cells within CD40-B cell preparations precludes that donor-derived T cells are included in CFSE-profiles of responder T cells, which would lead to wrong PF calculations.

The second improvement is the use of CFSE-dilution in combination with software that calculates PF as a read-out technique. Detection of responding cells by this technique is not dependent on the number of progeny cells in each individual culture, which in LDA may lead to underestimation of PF when small clones of progeny cells remain undetected [17], [24], [25], [46]. Moreover, estimated PF are not negatively influenced by “preparation-induced cell death” of responder T cells at the beginning of the cultures, because PF are calculated from the sum of precursors present at the end of the cultures instead from cell input at the start of the culture. We showed that allogeneic stimulations are prone to significant loss of responder T cells during the first 2 days of culture. Since dying cells do not respond, techniques calculating PF from numbers of cells present at the start of the culture, like LDA and ELISPOT, underestimate actual PF [38]. Conversely, the CFSE-MLR may overestimate PF if more non-proliferating cells compared to proliferating cells die during culture. It is difficult to analyze whether non-proliferating cells die, but the appearance of a plateau phase of PF between day 5 and 6 of culture indicates that at least during this last phase of the cultures no preferential death of non-proliferating T cells occurs.

ELISPOT also underestimates PF by ignoring allo-reactive T cells that do not secrete the particular cytokine detected. Indeed, we observed that not all T-cells that responded to allogeneic CD40-B cells by cell division (as determined by CFSE-dilution) produced IFN-γ. In addition, our data show that the numbers of IFN-γ producing T cells detected in ELISPOT are considerably lower compared to those detected by flowcytometry in CFSE-MLR, demonstrating the lower sensitivity of the ELISPOT-technique. In contrast to short-term ELISPOT assays which detect only the rapidly reacting allo-reactive memory T cells [23], the CFSE-MLR detects allo-reactive precursors in memory and naïve T cells simultaneously. Because the allo-reactive repertoire contains both naive and memory T cells [10], [47], [48], we judge that simultaneous quantification of both subsets is an advantage of the current technique compared to ELISPOT. Together, these differences may explain why published PF of donor-specific T cells in human organ transplant recipients detected by LDA [8], [9], [10], [13], [49], [50] or ELISPOT [22], [23], [51], [52], [53] are significantly lower compared to those observed in this study with the CFSE-MLR. Importantly, the use of CD40-B cells as stimulators to detect allogeneic T-cell responses [54], and CFSE-dilution as a technique to measure the proliferative response of T cells to allo-antigens [17], [24], [25], [46], [47] have both been described, but to our best knowledge these techniques have never been combined.

It is generally assumed that recipient T-cell responses against directly presented donor allo-antigen peak shortly after transplantation [2], but this has never been formally proven in humans. So far, the only other study that quantified donor-specific T-cell responses longitudinally after human organ transplantation [51] actually found a nadir in frequencies of donor-specific T cells at 1 week after kidney transplantation using IFN-γ ELISPOT. We observed, after correction for changes in responses to 3^rd^ party allo-antigens, a significant, but modest, increase of circulating donor-specific CD4^+^ and CD8^+^ T-cell numbers immediately after LTx, followed by a decrease to levels equal to numbers of T cells responding to 3^rd^ party allo-antigens within the first year. This increase was not due to variations in immunosuppression, because it was corrected for variations in 3^rd^ party responses. Moreover, the highest blood trough concentrations of the calcineurin inhibitors CsA and Tacrolimus were observed in these patients at 1 week after LTx, after which they gradually decreased (data not shown), indicating that the increase of numbers of donor-specific T cells during the first week after LTx occurred despite high levels of immunosuppressive drugs, and that the decrease afterwards was not the consequence of increasing levels of immunosuppressive drugs. The observed increase in T-cell PF reacting to 3^rd^ party allo-antigens, which was smaller compared to the increase in donor-specific T-cell PF, may be explained by partial overlap in HLA between donor and 3^rd^ party stimulator cells spleen CD40-B cells. Due to limited availability of banked splenocytes, complete HLA mismatching between donor and 3^rd^ party stimulators was not always possible. Our results are congruent with recent observations in mice showing a significant increase in direct pathway donor-specific T-cells shortly after transplantation [55], which is probably related to migration of donor-derived dendritic cells from the graft into the recipient [7].

With LDA, it has repeatedly been shown that after organ transplantation in humans, numbers of circulating donor-specific T cells decrease below pre-transplant values and below numbers of T cells responding to 3^rd^ party allo-antigens [8], [9], [10], [11], [13], [22], [39]. Donor-specific hypo-responsiveness was already observed at 1 month after LTx [13]. This phenomenon was attributed to induction of anergy in donor-specific T cells [56] or to suppression exerted by CD4^+^Foxp3+ regulatory T cells [11]. Of notice, donor-specific hypo-responsiveness in cytotoxic T-cell PF detected by LDA after lung transplantation, could not be confirmed using flow-cytometric detection of CD8^+^ T-cell activation and IFN-γ production [57]. Therefore, our observation that no loss of donor-specific T-cells occurs after LTx is probably due the higher sensitivity of the CFSE-MLR compared to LDA. Re-stimulation of CFSE-labeled recipient T cells during the last day of culture with CD40-B cells of the same donor allowed quantification of effector function (IFN-γ production) of responding T cells. The data revealed that there is a decrease in the proportions of recipient T-cells producing IFN-γ at 1 year after LTx compared to pre-LTx values. This decrease, however, occurred both upon stimulation with donor-derived and with 3^rd^ party-derived CD40-B cells, demonstrating that no donor-specific loss of T-cell functionality occurs after LTx. We suppose that the non-specific decrease in the capacity of T cells to produce IFN-γ after LTx is probably due to the continuous treatment with immunosuppressive medication. In summary, our data show that no specific loss of circulating donor-specific T-cell clones occurs during the first year after LTx, that their capacity to expand in response to donor allo-antigens is undisturbed, and that they are still able to mount effector function although at a lower level because of a general impairment of T-cell effector function. These conclusions are consistent with those of Kusaka et al [58], who showed high levels of donor HLA-specific T-cell clonotype mRNAs in PBMC late after renal transplantation. Both studies imply that T cells which recognize donor allo-antigens via the direct pathway remain present in the recipient circulation for at least one year after transplantation. Whether donor-specific hypo-responsiveness might develop later after transplantation will be subject of a future study.

Of the 18 patients studied in the current paper, 4 experienced one or more episodes of acute rejection, occurring between day 10 and day 60 after LTx. The data show some tendency of higher pre-LTx CD3+ and CD8+ donor-specific T-cell PF in the patients which developed acute rejection, but this difference is statistically not significant. The present study was not designed for studying differences between rejectors and non-rejectors, neither powered for that purpose. Associations between donor-specific T-cell PF and acute or chronic rejection will be the subject of a later study with larger numbers of patients.

In conclusion, by using a novel technical approach, we showed for the first time an increase of donor-specific T-cell frequencies shortly after LTx in humans. In addition, we observed that T cells reacting to donor allo-antigens presented via the direct pathway remain present in the recipient circulation for at least 1 year after transplantation.
